# “It Works for Me”: Pseudotherapy Use is Associated With Trust in Their Efficacy Rather Than Belief in Their Scientific Validity

**DOI:** 10.3389/ijph.2022.1604594

**Published:** 2022-09-16

**Authors:** Gregorio Segovia, Belén Sanz-Barbero

**Affiliations:** ^1^ Department of Physiology, Faculty of Medicine, Universidad Complutense de Madrid, Madrid, Spain; ^2^ Joint Research Institute National Distance Education University and Health Institute Carlos III (IMIENS), Madrid, Spain; ^3^ Epidemiology and Statistics Department, National School of Public Health, Carlos III Institute of Health, Madrid, Spain; ^4^ CIBER Epidemilogy and Public Health (CIBERESP), Madrid, Spain

**Keywords:** Spain, gender, health, pseudotherapy, patient attitudes, patient beliefs

## Abstract

**Objectives:** To identify how perceptions, attitudes, and beliefs towards pseudotherapies, health, medicine, and the public health system influence the pseudotherapy use in Spain.

**Methods:** We carried out a cross-sectional study using the Survey of Social Perception of Science and Technology-2018 (5,200 interviews). Dependent variable: ever use of pseudotherapies. Covariables: attitude towards medicine, health and public health system; perceived health; assessment of the scientific character of homeopathy/acupuncture. The association was estimated using prevalence ratios obtained by Poisson regression models. The model was adjusted for age and socioeconomic variables.

**Results:** Pseudotherapy use was higher in women (24.9%) than in men (14.2%) (*p* < 0.001). The probability of use in men (*p* < 0.001) and women (*p* < 0.001) increases with the belief in pseudotherapies’ usefulness. Among men, a proactive attitude (reference: passive) towards medicine and health (RP:1.3), and a negative (reference: positive) assessment of the quality of the public health system increased use-probability (RP:1.2). For women, poor health perceived (referencie: good) increased likelihood of use (RP:1.2).

**Conclusion:** Pseudotherapy use in Spain was associated with confidence in its usefulness irrespective of users’ assessment of its scientific validity.

## Introduction

A pseudotherapy is considered to be “a substance, product, activity or service with a purported health purpose that does not have support from scientific knowledge or evidence guaranteeing its efficacy and safety” [1]. The use of pseudotherapies is quite accepted in Europe—according to the *European Social Survey, Round 7* (edition 2.0, 2014), 25.9% of the general population had used them in the last 12 months [2]. In Spain, their use is also prevalent—despite high levels of trust in conventional medicine and health professionals—ranging between 17.2% in the last 12 months [2] and 19.6% for having ever used them [3]. The sociodemographic profile of the pseudotherapy user in Spain is that of a middle/upper class woman, with higher-level university studies and a progressive political ideology [4]. The use of pseudotherapies can imply genuine health risks because this use is based on misleading advertising about the expected therapeutic effects, and this can favour the delay or substitution of conventional treatments, or even reduce the effectiveness of the latter [1].

Pseudotherapy use has been linked to negative experiences with conventional evidence-based medicine, ranging from a negative doctor-patient interaction to the perception of conventional medicine’s ineffectiveness, through to the side effects of conventional treatments [5–7]. In this way, the use of pseudotherapies would be motivated by being perceived as risk-free, in part because their development has not been influenced by the interests of pharmaceutical companies [8, 9]. However, motivation for pseudotherapy use is not simply derived from a rejection of conventional medicine or a poor perception of health systems [10]. On the contrary, pseudotherapy users are defined as proactive subjects with the ability to choose their treatments [5, 6, 9] and, for this reason, they carry out an individualized search for those types of treatments that they believe will be effective for them [7].

On the other hand, some studies have shown that the use of pseudotherapies is motivated by rejection of science and conventional medicine, especially in relation to the perception of their limitations and side effects [11–13]. However, other studies have suggested that pseudotherapy users have coexisting beliefs in the efficacy of both these and conventional medicine—which is why they use pseudotherapies as complementary treatments [10, 14, 15]. This could be explained by pseudotherapies’ scientific appearance, which is constructed, at least in part, through the way they are presented in the media [10, 16]. The appearance of scientific validity is supported by the recommendations of some doctors and, for pseudotherapies such as homeopathy, they are dispensed in pharmacies together with drugs that have had their efficacy shown scientifically [10].

The objective of the present study was to identify how the following factors have influenced pseudotherapy use in Spain:

- Attitude towards medicine and health and evaluation of the public health system, as well as perceived health.
- Confidence in the efficacy of pseudotherapies and assessment of their scientific validity.

To do this, we carried out a cross-sectional study of the Survey of Social Perception of Science and Technology 2018 [3] of the Spanish Foundation for Science and Technology (FECYT). This 9th edition of the survey included a question on the use of pseudotherapies for the first time. It also gathered data on the population’s perception of pseudotherapies, and opinions on science and medicine – together with sociodemographic contextual variables.

## Methods

### Data Source

The survey [3] includes a total of 5,200 non-institutionalized people who had been resident in Spain (Peninsula, Balearic Islands and Canary Islands) at least 5 years. The autonomous cities of Ceuta and Melilla were not included. A multi-stage, stratified sampling was carried out, with random and proportional selection of primary units (municipality) and secondary (sections). The tertiary units (individuals) were identified by random routes and sex and age quotas. To obtain nationally representative data, weighting coefficients were included so that the interviews carried out in each of the Autonomous Communities were adjusted to the real population weightings. The information was collected through computer-assisted home interviews (CAPI- Computer - Assisted Personal Interviewing). This fieldwork took place during the period 14 May–2 July 2018.

### Variables

#### Dependent Variables

The dependent variable analysed was the use of pseudotherapies, collected under the question “*Have you ever used alternative treatments such as homeopathy or acupuncture?*”

#### Exposure Variables

• Interest in/knowledge of Medicine and Health: low [2–4]; medium [5–7]; high [8–10]. Variable constructed from the sum of the following Likert-type variables (range 1–5): 1) *Now, I would like to know if you are very little, little, somewhat, quite or very interested in the following topics: medicine and health. 2*) *Now, I would like you to tell me whether you consider yourself: very little, a little, somewhat, quite or very informed about each of these same topics: medicine and health.*
• Perceived health: *In general, would you say that your health is ...? good/very good; fair/bad/very bad*
• Quality of the health system: *In general, how would you rate the quality of the public health system: good/rather good; rather bad/bad*
• Confidence in homeopathy and acupuncture: low [2–4]; mean [5–7]; high [8–10]. Variable constructed from the summation of the following Likert-type variables (range 1–5): *Of the following practices, please tell me if you trust a lot, a lot, something, little or not at all in its usefulness for health and general well-being:* 1) *acupuncture;* 2) *homeopathy*
• Scientific validity of homeopathy/acupuncture: low [2–4]; medium [5–7]; high [8–10]. Variable constructed from the summation of the following Likert-type variables (range 1–5): *To what degree do you think the following practices are scientific valid, using a scale where the number one means that it is* “*not at all scientifically valid*” *and the number 5 means* “*totally scientifically valid*”*:* 1) *acupuncture;* 2) *homeopathy*

In addition, they included sociodemographic variables, specifically: age categorized by decades (with the exception of the last group which included 65 and over); educational level of the person interviewed (primary or lower; secondary; higher); and family income (above average/average/below average/ns/nc).

### Statistical Analysis

First, we carried out a descriptive study. We compared the distribution of pseudotherapy use according to the variables previously described (Chi-square test). Then, using Poisson regressions with robust variance [17], crude prevalence ratios for pseudotherapy use were calculated and adjusted by the exposure variables and covariates previously noted. In order to analyse whether pseudotherapy use differs between men and women, possible interactions by sex were also explored. As interactions were found, the results are presented with stratification by sex. All analyses were performed using the weights included in the survey. The analysis was carried out using the Stata 15.0 program.

## Results

Of the 5,200 people surveyed, information on pseudotherapy use was obtained from 5,183 (nc = 17). 48.7% of the sample were men and 51.3% women. The prevalence of pseudotherapy use, e.g., homeopathy or acupuncture, at some time in their lives was 19.6%—higher in women than in men (24.9% vs 14.2%, *p* < 0.001). Of the participants who reported the use of pseudotherapies, 26.3% used them as an alternative therapy and 73.5% as a complementary therapy.

Table 1 describes the prevalence of pseudotherapy use according to the covariates studied. In both sexes, use prevalence is significantly higher in people who answered that they had a high level of interest in and knowledge of medicine and health (Prev_men: 19.7%; Prev___women: 29.9%), in those who have a high confidence in the usefulness of homeopathy and acupuncture Prev_men: 34.1%; Prev_women: 48.2%), as well as in people who perceive that these pseudotherapies have a high level of scientific validity (Prev_men: 23.9%; Prev_women: 37.4%)

**TABLE 1:** Pseudotherapy use Prevalence. Spanish Survey of Social Perception of Science and Technology, Spain, 2018.

Have you ever used alternative treatments such as homeopathy or acupuncture?										
Covariables	Male					Women				
	Yes		Total		*p*	Yes		Total		*p*
	(n)^a^	(%)	(n)^a^	(%)		(n)^a^	(%)	(n)^a^	(%)	
Interest and knowledge in medicine and health										
Low/Medium	204	11.7	1751	69.6	<0.001	310	20.9	1468	55.8	<0.001
High	150	19.7	763	30.4		348	29.7	1165	44.2	
Quality of the health system										
Very good/good	233	13.3	1764	69.9	0.025	451	25.0	1809	68.0	0.836
Bad/very bad	126	16.5	759	30.1		209	24.3	851	32.0	
Confidence in pseudotherapies										
Low	35	4.2	847	39.3	<0.001	50	7.1	707	30.4	<0.001
Average	151	17.0	888	41.2		251	25.6	979	42.0	
High	144	34.1	422	19.6		310	48.2	643	27.6	
Pseudotherapies’ scientifc validity										
Low/Medium	108	9.8	1102	47.7	<0.001	207	19.1	1084	44.1	<0.001
Medium	149	17.9	835	36.2		246	27.2	904	36.8	
High	89	24.0	372	16.1		175	37.7	468	19.1	
Perceived health										
Very good/good	277	13.8	2016	79.9	0.156	488	23.4	2085	78.4	0.001
Fair/Bad/Very bad	82	16.1	506	20.1		172	29.7	575	21.6	
Age										
15 to 24	33	8.1	407	16.1	<0.001	53	12.3	431	16.2	<0.001
25 to 34	62	12.4	499	19.8		118	22.7	519	19.5	
35 to 44	88	18.9	467	18.5		142	28.5	498	18.7	
45 to 54	64	17.0	377	14.9		121	30.6	397	14.9	
55 to 64	50	16.2	308	12.2		110	33.5	330	12.4	
65 and over	62	13.4	465	18.4		116	23.7	485	18.2	
Level of studies completed										
Primary or lower	40	10.6	379	15.0	<0.001	83	21.2	392	14.7	0.001
Secondary	224	13.5	1664	66.0		394	23.7	1665	62.6	
Higher education	94	19.5	479	19.0		184	30.4	604	22.7	
Family Income										
Higher than average	153	17.3	886	35.1	<0.001	245	29.9	819	30.8	0.001
Around average	95	13.1	727	28.8		197	23.6	834	31.3	
Lower than average	63	15.6	407	16.1		115	23.0	504	18.9	
missing	48	9.4	503	19.9		105	20.7	505	19.0	
Total	358	14.2	2522	48.67		660	24.8	2660	51.33	

a Weighed value.

In men, use prevalence is significantly higher among those who consider that public health system quality is poor/very poor (16.6%). In women, this prevalence is higher in those who report poor perceived health (Prev_women: 29.9%).

Table 2 shows the variables associated with the use of alternative treatments such as homeopathy or acupuncture. Regardless of the sociodemographic characteristics of the people interviewed, in both sexes the probability of using alternative treatments was greater as confidence in their usefulness for general health and well-being increased (*p* < 0.001).

**TABLE 2:** Variables associated with the use of pseudotherapies. Spanish Survey of Social Perception of Science and Technology, Spain, 2018.

Variables	Men			Women		
	RP	IC (95%)		RP	IC (95%)	
Interest and knowledge in medicine and health						
Low/Medium	1			1		
High	1.33	1.09	1.62	1.09	0.96	1.25
Perceived health						
Very good/good	1.00			1.00		
Fair/Bad/Very bad	1.00	0.78	1.30	1.23	1.05	1.42
Quality of the health system						
Very good/good	1.00			1.00		
Bad/very bad	1.24	1.01	1.52	0.98	0.85	1.13
Confidence in pseudotherapies						
Low	1.00			1.00		
Average	4.15	2.77	6.22	3.70	2.73	5.03
High	8.03	5.28	12.21	6.62	4.88	8.99
Pseudotherapies’ scientifc validity						
Low	1.00			1.00		
Medium	1.06	0.82	1.36	0.96	0.81	1.13
High	1.02	0.77	1.34	1.05	0.88	1.25

RP adjusted by age, family income, educational level of the person interviewed.

In men, use probability increased in those with a medium/high level of knowledge and interest in medicine and health (PR (59% CI): 1.33 (1.09; 1.62)] (ref: low interest), as well as in those who have a negative perception of the quality of the public health system [PR (59% CI): 1.24 (1.01; 1.52)] (ref: positive). In the case of women, the probability of using alternative treatments is greater in those with poor perceived health (PR (59% CI): 1.23 (1.05, 1.42)] (ref: good). The level of scientific validity attributed to homeopathy and acupuncture was not associated with the probability of using alternative therapies, in either sex.

## Discussion

The results of the EPSCYT 2018 analysis show that pseudotherapy-use prevalence at some point in their lives is higher in women (24.9%) than in men (14.2%) and that the probability of using pseudotherapies increases with increasing confidence in their usefulness—regardless of users’ assessment of their scientific validity. Added to this, several factors influence the probability of men and women’s pseudotherapy use: perceived poor health increases this probability in women, while a positive attitude towards medicine and health and a negative assessment of quality of the public health system increases it in men.

As we have indicated, pseudotherapy-use prevalence is higher in women (24.9%) than in men (14.2%). In the full sample, use prevalence at some point during their lives is 19.6%. This data is in line with that obtained in the 2014 *European Social Survey* for Spain: 17.2% in the last 12 months [2]. The analysis in that survey also shows that the probability of using pseudotherapies is higher in women across the whole sample [2]. For its part, the barometer of the Sociological Research Centre (CIS), which provides data on the use of pseudotherapies in Spain in the last 12 months in a disaggregated manner, shows a homeopathy-use prevalence of 5% [4]. In accordance with our analysis of the EPSCYT 2018, the CIS-survey analysis indicates that the majority of homeopathy users are women (66.1%) [4]. Finally, the analysis of three editions of the National Health Survey (2011, 2014 and 2017) showed a homeopathy-use prevalence in the prior 2 weeks of 1.06%, with higher use-probability in women than in men [18]. The higher prevalence of pseudotherapy use in women could be non-specific since the *European Social Survey* from 2014 shows that women use primary care and medical specialists more than men, which could be associated with the fact that they also describe a greater unmet need for medical care [19].

The main result of this study is that pseudotherapy use was associated with confidence in their usefulness irrespective of users’ assessment of their scientific validity. Use prevalence is significantly higher in both the people who have high confidence in the usefulness of homeopathy and acupuncture as well as those who perceive that these pseudotherapies have a higher level of scientific validity. However, the multivariate regression model showed that users’ assessment of the therapies’ scientific validity is explained by their confidence in those therapies’ usefulness. Among other causes, this confidence in the usefulness of pseudotherapies may be motivated by an intuitive and uncritical acceptance of the correlation between their use and the perception of efficacy (illusion of causality), which is reinforced by the tendency to selectively remember only the results that lead to improvements [20–22]. Initially, a positive perception of pseudotherapies’ usefulness may arise from exposure to very convincing personal narratives or biased sources such as online forums and social networks, although it can also be derived from the recommendation of medical professionals themselves [6, 22, 23]. But as Atwell et al. suggest, having expectations of the perceived efficacy of pseudotherapies being fulfilled would increase the users’ confidence in their ability to make decisions regarding their health (illusion of control), thus reinforcing the decision to use them again [9]. Recently Ciocănel et al. have shown how the narrative of the apparent efficacy of pseudotherapies like homeopathy is the main justification given by users to legitimize their use [24]. However, the EPSCYT 2018 does not include a question on the satisfaction of previous use of pseudotherapies. This would have been helpful in determining the role of perceived positive experiences in confidence about their usefulness.

On the other hand, use-prevalence is significantly higher in the people who report having a high level of interest in and knowledge of medicine and health, although the multivariate analysis showed that this variable is associated with the use of pseudo-therapies only in men. This result is in line with the results of the analysis of the previous EPSCYT survey, in which a positive association was observed between interest in science and confidence in the effectiveness of pseudotherapies such as homeopathy and acupuncture [10]. Lobera and Rogero-García propose that this association could be explained by appearance of scientific validity pseudotherapies have, based on their presentation as effective medical treatments by the media. In addition, they are recommended by some doctors and, in the case of homeopathy, may be dispensed in pharmacies together with medicines that have been shown to be effective through rigorous scientific testing [10]. In this way, a proportion of pseudotherapy users would maintain belief in the efficacy of both these and conventional medicine, which would lead to the use of pseudotherapies as complementary treatments [10, 14, 15].

Another factor associated with pseudotherapy use in men is the assessment of the quality of the public health system: a poor or very poor assessment was associated with greater use. On the other hand, pseudotherapy use in women was associated with perceived poor health. Various studies have shown that pseudotherapy use can be motivated by negative experiences with conventional medicine, for instance: no improvement in symptoms, poor doctor-patient interaction and treatments’ side effects [6, 7, 24]. This would lead to the search for pseudotherapies as alternative or complementary treatments, especially considering the perception of such treatments as low risk [8, 9, 23]. In addition, pseudotherapy users are defined as active subjects who want to have control over their health and the use of alternative treatments would provide them with a feeling of empowerment [6, 7, 9].

Finally, the results of this study should be taken into account in the implementation of strategies to protect citizens against pseudotherapies [1]. One of these possible strategies would be to disseminate accurate information on pseudotherapies’ effectiveness to both the population and health-related interest groups, based on the “information deficit model” [22]. For these campaigns to be effective, it is important to take into account the psychological factors that induce belief in pseudotherapies’ effectiveness, such as the illusion of causality [20, 25] and the illusion of control [26] and show the mechanisms by which an ineffective intervention can be correlated with apparent efficacy [27, 28]. The cultural factors that help to legitimize pseudotherapies and their use should also be considered, especially narratives about their efficacy [24]. Finally, it is necessary to note the differing factors associated with pseudotherapy-use of in men and women and the higher use prevalence in women [19].

### Limitations

This study must be interpreted with due consideration given to its limitations. The cross-sectional design does not allow us to establish causal relationships; however, it is an adequate design to estimate prevalences while at the same time allowing us to identify which factors could increase pseudotherapy-use probability. The low use of pseudotherapies among those who have low confidence in pseudosciences, reduces the precision of association measured, but does show a consistent positive trend in both sexes. Given that the percentage of people who did not provide information on family income was high, in order to avoid loss of information, the adjustment for this variable included missing values as an independent category. To reduce the possible lack of precision in the adjustment for socioeconomic level, this was adjusted for using the educational level of the people interviewed. Finally, the question used as the dependent variable refers to the use of two pseudotherapies, homeopathy and acupuncture, that may be seen as different regarding their scientific validity. This may have it made difficult for the participants of the survey to answer the question.

### Conclusion

Determining the factors associated with pseudotherapy use is of great importance since these can imply a real risk to the health of individuals [1]. The results of the study indicate that pseudotherapy use is associated with confidence in their utility regardless of users’ assessment of their scientific validity. Previous studies analysing earlier EPSCYT surveys found that the apparent scientific validity with which pseudotherapies are presented could play an important role in their use [10]. However, our results suggest that the perception of pseudotherapies’ scientific validity is rather an *a posteriori* justification of use motivated by their apparent efficacy. This finding is relevant for developing strategies aimed at reducing pseudotherapy use, acting both at the level of their presentation and marketing, and at the level of the health personnel who sometimes recommend their use.

## References

[1] MINISTERIO DE SANIDAD, CONSUMO Y BIENESTAR SOCIAL. Plan for Protecting Health against Pseudotherapies (2018 ). https://www.lamoncloa.gob.es/serviciosdeprensa/notasprensa/sanidad/Documents/141118PlanProtecci%C3%B3n_pseudoterapias.pdf (Accessed April 9, 2020).

[2] Kemppainen LM Kemppainen TT Reippainen JA Salmenniemi ST Vuolanto PH. Use of Complementary and Alternative Medicine in Europe: Health-Related and Sociodemographic Determinants. Scand J Public Health (2018) 46:448–55. 10.1177/1403494817733869 28975853 PMC5989251

[3] Encuesta de Percepción Social de la Ciencia (EPSCYT). INFORME DE RESULTADOS (2018 ). https://www.fecyt.es/sites/default/files/news/attachments/2018/11/epscyt2018_informe_0.pdf (Accessed February 20, 2020).

[4] Cano-Orón L Mendoza-Poudereux I Moreno-Castro C. [Sociodemographic Profile of the Homeopathy User in Spain].. Aten Primaria (2019) 51:499–505. 10.1016/j.aprim.2018.07.006 30262221 PMC6836979

[5] Evans MA Shaw ARG Sharp DJ Thompson EA Falk S Turton P Men with Cancer: Is Their Use of Complementary and Alternative Medicine a Response to Needs Unmet by Conventional Care? Eur J Cancer Care (2007) 16:517–25. 10.1111/j.1365-2354.2007.00786.x 17944766

[6] Keene MR Heslop IM Sabesan SS Glass BD. Complementary and Alternative Medicine Use in Cancer: A Systematic Review. Complement Ther Clin Pract (2019) 35:33–47. 10.1016/j.ctcp.2019.01.004 31003679

[7] MacArtney JI Wahlberg A. The Problem of Complementary and Alternative Medicine Use Today: Eyes Half Closed? Qual Health Res (2014) 24:114–23. 10.1177/1049732313518977 24406483

[8] Blanco F Barberia I Matute H. The Lack of Side Effects of an Ineffective Treatment Facilitates the Development of a Belief in its Effectiveness. PLOS ONE (2014) 9:e84084. 10.1371/journal.pone.0084084 24416194 PMC3885525

[9] Attwell K Ward PR Meyer S Rokka PJ Leask J. Do-it-yourself”: Vaccine Rejection and Complementary and Alternative Medicine (CAM). Soc Sci Med (2018) 196:106–14. 10.1016/j.socscimed.2017.11.022 29175699

[10] Lobera J Rogero-García J. Scientific Appearance and Homeopathy. Determinants of Trust in Complementary and Alternative Medicine. Health Commun (2021) 36:1278–85. 10.1080/10410236.2020.1750764 32285701

[11] Beyerstein BL. Alternative Medicine and Common Errors of Reasoning. Acad Med (2001) 76:230–7. 10.1097/00001888-200103000-00009 11242572

[12] Conboy L Kaptchuka TJ Eisenberg DM Gottlieb B Acevedo-Garcia D. The Relationship between Social Factors and Attitudes toward Conventional and CAM Practitioners. Complement Ther Clin Pract (2007) 13:146–57. 10.1016/j.ctcp.2006.12.003 17631257

[13] Shmueli A Igudin I Shuval J. Change and Stability: Use of Complementary and Alternative Medicine in Israel: 1993, 2000 and 2007. Eur J Public Health (2011) 21:254–9. 10.1093/eurpub/ckq023 20375024

[14] Stoneman P Sturgis P Allum N Sibley E. Incommensurable Worldviews? Is Public Use of Complementary and Alternative Medicines Incompatible with Support for Science and Conventional Medicine? PLoS One (2013) 8:e53174. 10.1371/journal.pone.0053174 23382836 PMC3559728

[15] Thomas K Coleman P. Use of Complementary or Alternative Medicine in a General Population in Great Britain. Results from the National Omnibus Survey. J Public Health (2004) 26:152–7. 10.1093/pubmed/fdh139 15284318

[16] Lopera-Pareja EH Cano-Orón L. Media’s Portrayal of CAM: Exploring 40 Years of Narratives and Meanings in Public Discourse. Journalism (2021) 146488492098540. (in press). 10.1177/1464884920985407

[17] Barros AJ Hirakata VN. Alternatives for Logistic Regression in Cross-Sectional Studies: an Empirical Comparison of Models that Directly Estimate the Prevalence Ratio. BMC Med Res Methodol (2003) 3:21. 10.1186/1471-2288-3-21 14567763 PMC521200

[18] Pinilla J Rodríguez-Caro A. Differences in Healthcare Utilisation between Users and Non-users of Homeopathic Products in Spain: Results from Three Waves of the National Health Survey (2011-2017). PloS ONE (2019) 14:e0216707. 10.1371/journal.pone.0216707 31083699 PMC6513046

[19] Huijts T Stornes P Eikemo TA Bambra C The HiNews Consortium. The Social and Behavioural Determinants of Health in Europe: Findings from the European Social Survey (2014) Special Module on the Social Determinants of Health. Eur J Public Health (2017) 27:55–62. 10.1093/eurpub/ckw231 28355646

[20] Matute H Yarritu I Vadillo MA. Illusions of Causality at the Heart of Pseudoscience. Br J Psychol (2011) 102:392–405. 10.1348/000712610x532210 21751996

[21] Lilienfeld SO Ritschel LA Lynn SJ Cautin RL Latzman RD. Why Ineffective Psychotherapies Appear to Work: A Taxonomy of Causes of Spurious Therapeutic Effectiveness. Perspect Psychol Sci (2014) 9:355–87. 10.1177/1745691614535216 26173271

[22] MacFarlane D Hurlstone MJ Ecker UKH. Protecting Consumers from Fraudulent Health Claims: A Taxonomy of Psychological Drivers, Interventions, Barriers, and Treatments. Soc Sci Med (2020) 259:112790. 10.1016/j.socscimed.2020.112790 32067757

[23] Nissen N Schunder-Tatzber S Weidenhammer W Johannessen H. What Attitudes and Needs Do Citizens in Europe Have in Relation to Complementary and Alternative Medicine? Forsch Komplementmed (2006) 19:9–17. 10.1159/000342710 23883940

[24] Ciocặnel A Rughinis C Flaherty MG. Argumentative Time Work for Legitimizing Homeopathy: Temporal Reasons for the Acceptance of an Alternative Medical Practice. Time Soc (2021) 30:100–25. 10.1177/0961463X20962663

[25] Torres MN Barberia I Rodríguez-Ferreiro J. Causal Illusion as a Cognitive Basis of Pseudoscientific Beliefs. Br J Psychol (2020) 111:840–52. 10.1111/bjop.12441 32040216

[26] Yarritu I Matute H Vadillo MA. Illusion of Control: the Role of Personal Involvement. Exp Psychol (2014) 61:38–47. 10.1027/1618-3169/a000225 23948387 PMC4013923

[27] Blanco F Matute H. Diseases that Resolve Spontaneously Can Increase the Belief that Ineffective Treatments Work. Soc Sci Med (2020) 255:113012. 10.1016/j.socscimed.2020.113012 32387871

[28] Blanco F Moreno-Fernández MM Matute H. Are the Symptoms Really Remitting? How the Subjective Interpretation of Outcomes Can Produce an Illusion of Causality. Judgm Decis Mak (2020) 15:572–85.

